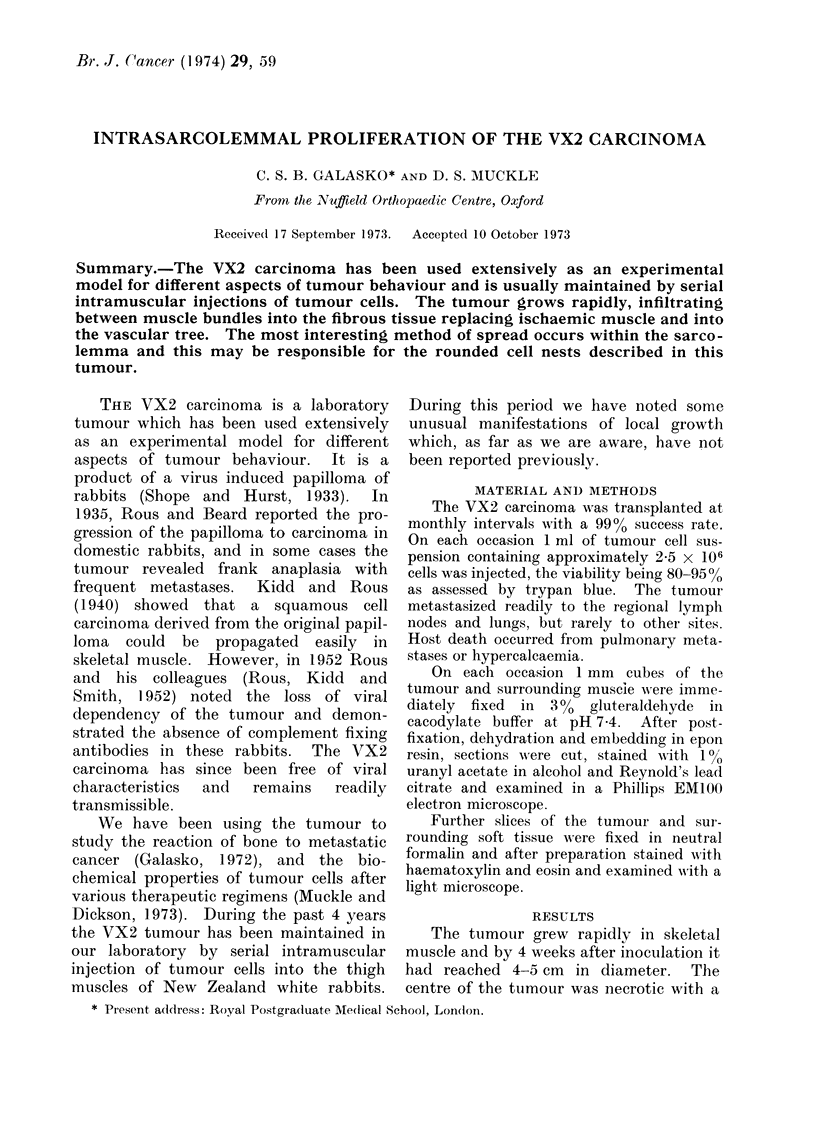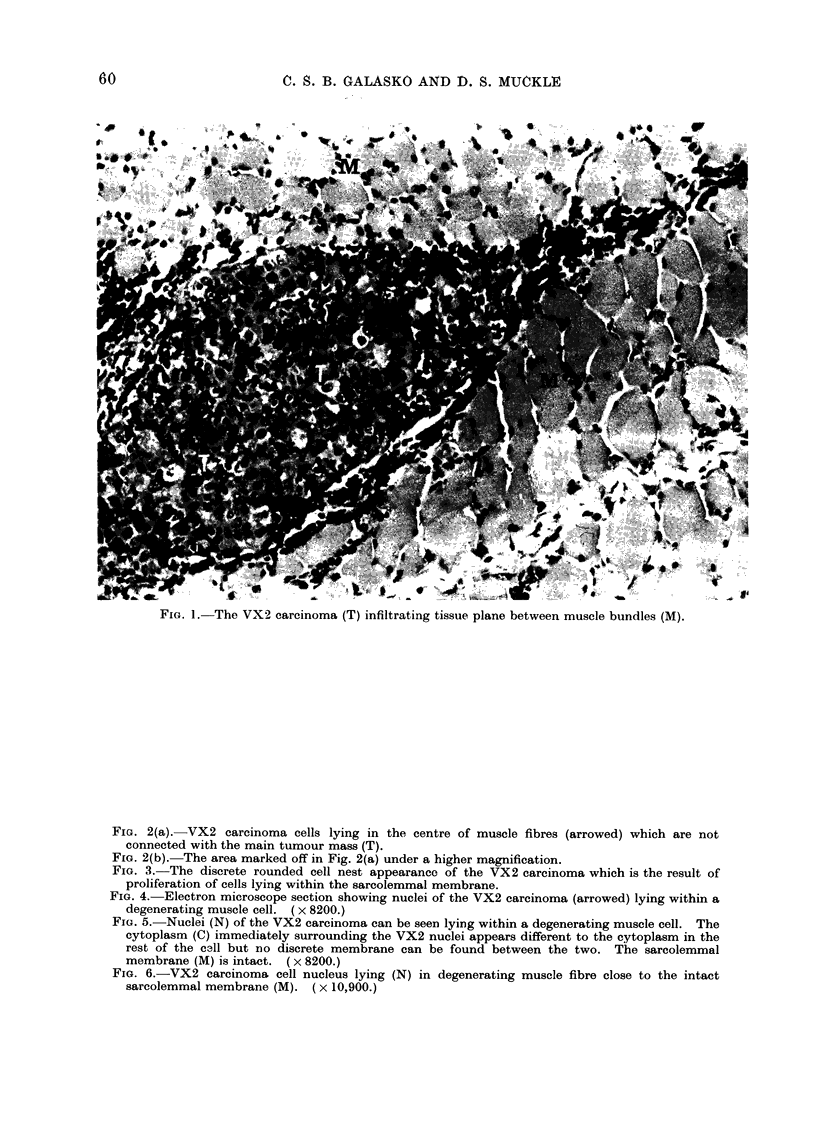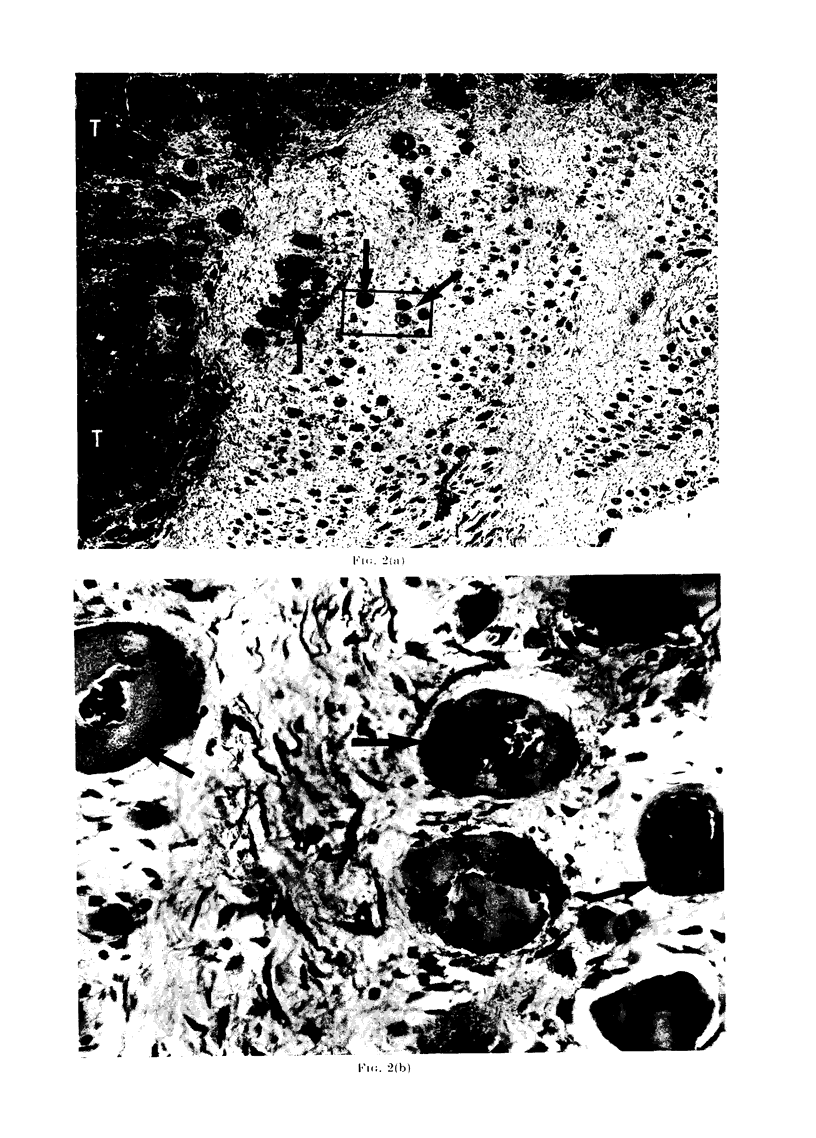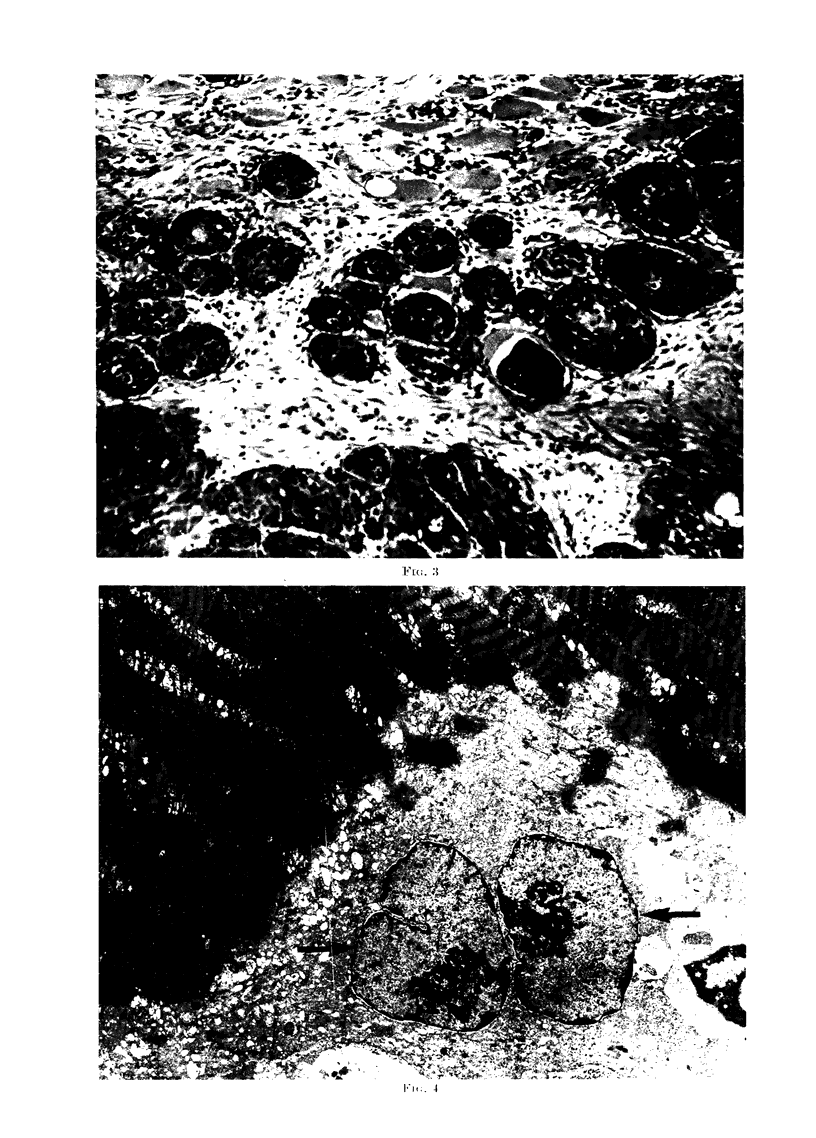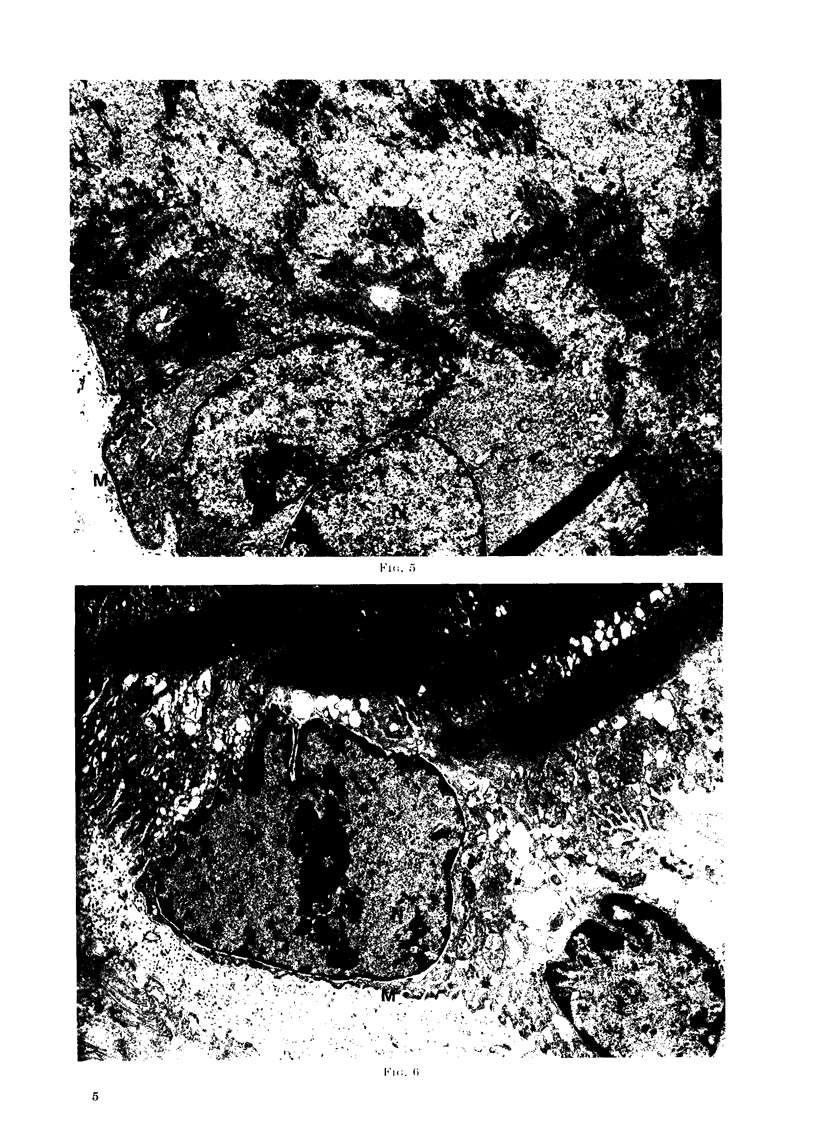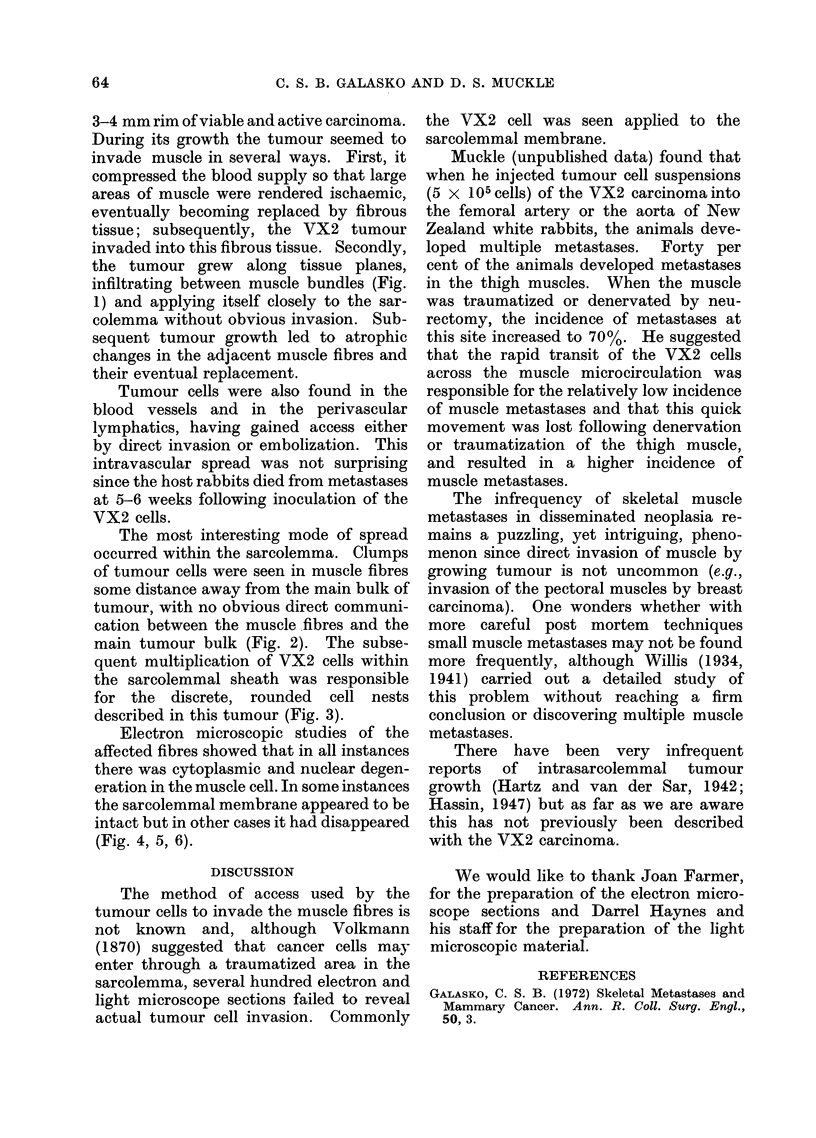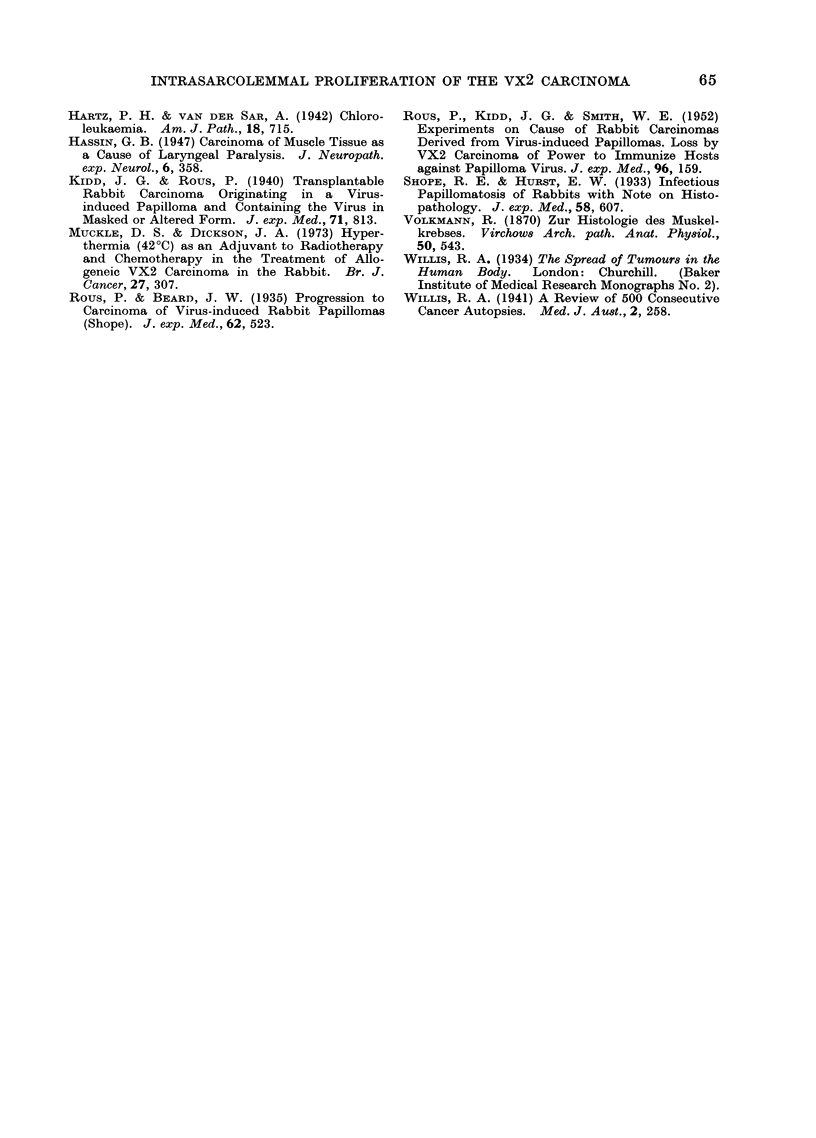# Intrasarcolemmal Proliferation of the VX2 Carcinoma

**DOI:** 10.1038/bjc.1974.7

**Published:** 1974-01

**Authors:** C. S. B. Galasko, D. S. Muckle

## Abstract

**Images:**


Br. J. Cancer (1974) 29, 59

### INTRASARCOLEMMAL PROLIFERATION OF THE VX2 CARCINOMA

C. S. B. GALASKO* AND D. S. MUCKLE

*From the Nuffield Orthopaedic Centre, Oxford*

Received 17 September 1973. Accepted 10 October 1973

**Summary.—The VX2 carcinoma has been used extensively as an experimental model for different aspects of tumour behaviour and is usually maintained by serial intramuscular injections of tumour cells. The tumour grows rapidly, infiltrating between muscle bundles into the fibrous tissue replacing ischaemic muscle and into the vascular tree. The most interesting method of spread occurs within the sarcolemma and this may be responsible for the rounded cell nests described in this tumour.**

THE VX2 carcinoma is a laboratory tumour which has been used extensively as an experimental model for different aspects of tumour behaviour. It is a product of a virus induced papilloma of rabbits (Shope and Hurst, 1933). In 1935, Rous and Beard reported the progression of the papilloma to carcinoma in domestic rabbits, and in some cases the tumour revealed frank anaplasia with frequent metastases. Kidd and Rous (1940) showed that a squamous cell carcinoma derived from the original papilloma could be propagated easily in skeletal muscle. However, in 1952 Rous and his colleagues (Rous, Kidd and Smith, 1952) noted the loss of viral dependency of the tumour and demonstrated the absence of complement fixing antibodies in these rabbits. The VX2 carcinoma has since been free of viral characteristics and remains readily transmissible.

We have been using the tumour to study the reaction of bone to metastatic cancer (Galasko, 1972), and the biochemical properties of tumour cells after various therapeutic regimens (Muckle and Dickson, 1973). During the past 4 years the VX2 tumour has been maintained in our laboratory by serial intramuscular injection of tumour cells into the thigh muscles of New Zealand white rabbits. During this period we have noted some unusual manifestations of local growth which, as far as we are aware, have not been reported previously.

### MATERIAL AND METHODS

The VX2 carcinoma was transplanted at monthly intervals with a 99% success rate. On each occasion 1 ml of tumour cell suspension containing approximately $2{\cdot}5 \times 10^6$ cells was injected, the viability being 80–95% as assessed by trypan blue. The tumour metastasized readily to the regional lymph nodes and lungs, but rarely to other sites. Host death occurred from pulmonary metastases or hypercalcaemia.

On each occasion 1 mm cubes of the tumour and surrounding muscle were immediately fixed in 3% gluteraldehyde in cacodylate buffer at pH 7·4. After postfixation, dehydration and embedding in epon resin, sections were cut, stained with 1% uranyl acetate in alcohol and Reynold's lead citrate and examined in a Phillips EM100 electron microscope.

Further slices of the tumour and surrounding soft tissue were fixed in neutral formalin and after preparation stained with haematoxylin and eosin and examined with a light microscope.

### RESULTS

The tumour grew rapidly in skeletal muscle and by 4 weeks after inoculation it had reached 4–5 cm in diameter. The centre of the tumour was necrotic with a

* Present address: Royal Postgraduate Medical School, London.

FIG. 1.—The VX2 carcinoma (T) infiltrating tissue plane between muscle bundles (M).

FIG. 2(a).—VX2 carcinoma cells lying in the centre of muscle fibres (arrowed) which are not connected with the main tumour mass (T).
FIG. 2(b).—The area marked off in Fig. 2(a) under a higher magnification.
FIG. 3.—The discrete rounded cell nest appearance of the VX2 carcinoma which is the result of proliferation of cells lying within the sarcolemmal membrane.
FIG. 4.—Electron microscope section showing nuclei of the VX2 carcinoma (arrowed) lying within a degenerating muscle cell. (× 8200.)
FIG. 5.—Nuclei (N) of the VX2 carcinoma can be seen lying within a degenerating muscle cell. The cytoplasm (C) immediately surrounding the VX2 nuclei appears different to the cytoplasm in the rest of the cell but no discrete membrane can be found between the two. The sarcolemmal membrane (M) is intact. (× 8200.)
FIG. 6.—VX2 carcinoma cell nucleus lying (N) in degenerating muscle fibre close to the intact sarcolemmal membrane (M). (× 10,900.)

Fig. 2(a)

Fig. 2(b)

Fig. 3

Fig. 4

FIG. 5

FIG. 6

3–4 mm rim of viable and active carcinoma. During its growth the tumour seemed to invade muscle in several ways. First, it compressed the blood supply so that large areas of muscle were rendered ischaemic, eventually becoming replaced by fibrous tissue; subsequently, the VX2 tumour invaded into this fibrous tissue. Secondly, the tumour grew along tissue planes, infiltrating between muscle bundles (Fig. 1) and applying itself closely to the sarcolemma without obvious invasion. Subsequent tumour growth led to atrophic changes in the adjacent muscle fibres and their eventual replacement.

Tumour cells were also found in the blood vessels and in the perivascular lymphatics, having gained access either by direct invasion or embolization. This intravascular spread was not surprising since the host rabbits died from metastases at 5–6 weeks following inoculation of the VX2 cells.

The most interesting mode of spread occurred within the sarcolemma. Clumps of tumour cells were seen in muscle fibres some distance away from the main bulk of tumour, with no obvious direct communication between the muscle fibres and the main tumour bulk (Fig. 2). The subsequent multiplication of VX2 cells within the sarcolemmal sheath was responsible for the discrete, rounded cell nests described in this tumour (Fig. 3).

Electron microscopic studies of the affected fibres showed that in all instances there was cytoplasmic and nuclear degeneration in the muscle cell. In some instances the sarcolemmal membrane appeared to be intact but in other cases it had disappeared (Fig. 4, 5, 6).

### DISCUSSION

The method of access used by the tumour cells to invade the muscle fibres is not known and, although Volkmann (1870) suggested that cancer cells may enter through a traumatized area in the sarcolemma, several hundred electron and light microscope sections failed to reveal actual tumour cell invasion. Commonly the VX2 cell was seen applied to the sarcolemmal membrane.

Muckle (unpublished data) found that when he injected tumour cell suspensions ($5 \times 10^5$ cells) of the VX2 carcinoma into the femoral artery or the aorta of New Zealand white rabbits, the animals developed multiple metastases. Forty per cent of the animals developed metastases in the thigh muscles. When the muscle was traumatized or denervated by neurectomy, the incidence of metastases at this site increased to 70%. He suggested that the rapid transit of the VX2 cells across the muscle microcirculation was responsible for the relatively low incidence of muscle metastases and that this quick movement was lost following denervation or traumatization of the thigh muscle, and resulted in a higher incidence of muscle metastases.

The infrequency of skeletal muscle metastases in disseminated neoplasia remains a puzzling, yet intriguing, phenomenon since direct invasion of muscle by growing tumour is not uncommon (*e.g.*, invasion of the pectoral muscles by breast carcinoma). One wonders whether with more careful post mortem techniques small muscle metastases may not be found more frequently, although Willis (1934, 1941) carried out a detailed study of this problem without reaching a firm conclusion or discovering multiple muscle metastases.

There have been very infrequent reports of intrasarcolemmal tumour growth (Hartz and van der Sar, 1942; Hassin, 1947) but as far as we are aware this has not previously been described with the VX2 carcinoma.

We would like to thank Joan Farmer, for the preparation of the electron microscope sections and Darrel Haynes and his staff for the preparation of the light microscopic material.

### REFERENCES

GALASKO, C. S. B. (1972) Skeletal Metastases and Mammary Cancer. *Ann. R. Coll. Surg. Engl.*, **50**, 3.

HARTZ, P. H. & VAN DER SAR, A. (1942) Chloroleukaemia. *Am. J. Path.*, **18**, 715.

HASSIN, G. B. (1947) Carcinoma of Muscle Tissue as a Cause of Laryngeal Paralysis. *J. Neuropath. exp. Neurol.*, **6**, 358.

KIDD, J. G. & ROUS, P. (1940) Transplantable Rabbit Carcinoma Originating in a Virus-induced Papilloma and Containing the Virus in Masked or Altered Form. *J. exp. Med.*, **71**, 813.

MUCKLE, D. S. & DICKSON, J. A. (1973) Hyperthermia (42°C) as an Adjuvant to Radiotherapy and Chemotherapy in the Treatment of Allogeneic VX2 Carcinoma in the Rabbit. *Br. J. Cancer*, **27**, 307.

ROUS, P. & BEARD, J. W. (1935) Progression to Carcinoma of Virus-induced Rabbit Papillomas (Shope). *J. exp. Med.*, **62**, 523.

ROUS, P., KIDD, J. G. & SMITH, W. E. (1952) Experiments on Cause of Rabbit Carcinomas Derived from Virus-induced Papillomas. Loss by VX2 Carcinoma of Power to Immunize Hosts against Papilloma Virus. *J. exp. Med.*, **96**, 159.

SHOPE, R. E. & HURST, E. W. (1933) Infectious Papillomatosis of Rabbits with Note on Histopathology. *J. exp. Med.*, **58**, 607.

VOLKMANN, R. (1870) Zur Histologie des Muskelkrebses. *Virchows Arch. path. Anat. Physiol.*, **50**, 543.

WILLIS, R. A. (1934) *The Spread of Tumours in the Human Body*. London: Churchill. (Baker Institute of Medical Research Monographs No. 2).

WILLIS, R. A. (1941) A Review of 500 Consecutive Cancer Autopsies. *Med. J. Aust.*, **2**, 258.